# Gingivitis in children and adolescents: epidemiological overview and associated factors—A narrative review

**DOI:** 10.3389/froh.2025.1675033

**Published:** 2025-10-10

**Authors:** Fatima Ezzahra Elgasmi, Kenza Maghous, Bouchra Badre

**Affiliations:** 1 Department of Odontological Sciences, Faculty of Medicine, Pharmacy and Dental Medicine, Sidi Mohammed Ben Abdellah University, Fez, Morocco; 2 Private Sector, Casablanca, Morocco; 3 Department of Pediatric Dentistry and Laboratory of Community Health, Epidemiology and Biostatistics, Faculty of Dental Medicine, Hassan II University, Casablanca, Morocco

**Keywords:** gingivitis, children, adolescents, epidemiology, risk factors, oral hygiene, prevention

## Abstract

**Background:**

Gingivitis is the most prevalent periodontal disease in children and adolescents. Although reversible, its high prevalence highlights a significant public health concern, especially in underserved populations.

**Methods:**

A narrative literature review was conducted using PubMed, Scopus, and Google Scholar to identify English and French studies published between 2014 and 2024. Inclusion criteria focused on epidemiological studies reporting prevalence, risk factors, and preventive strategies of gingivitis in pediatric populations.

**Results:**

Gingivitis prevalence among children and adolescents varies widely across countries, ranging from 20% to over 90%. Higher rates were observed in socioeconomically disadvantaged populations. Key risk factors include poor oral hygiene, sugar-rich diets, low parental education, orthodontic appliances, and limited access to preventive care.

**Conclusion:**

Gingivitis in pediatric populations remains a global concern. Multilevel strategies involving families, schools, and oral health professionals are essential to improve prevention, health education, and access to care in order to reduce disease burden.

## Background

Gingivitis, defined as inflammation of the gingival tissues without clinical attachment loss, is the most prevalent periodontal disease among children and adolescents (1). It is primarily caused by dental plaque accumulation at the gingival margin, leading to microbial dysbiosis. This shift—characterized by a reduction in gram-positive cocci and an increase in gramnegative anaerobic bacteria—triggers an inflammatory response that can result in gingival swelling, redness, and bleeding (2). Although gingivitis is considered reversible in its early stages, persistent inflammation may predispose individuals to periodontitis later in life (3).

Despite its clinical simplicity, pediatric gingivitis remains a major public health concern due to its high and variable prevalence worldwide. This variability is largely influenced by differences in diagnostic criteria, access to dental care, oral hygiene practices, and socioeconomic conditions (4–6). Recent epidemiological data from countries such as Nigeria (7), Myanmar (8), China (9), and Saudi Arabia (10) confirm that gingivitis is particularly prevalent in disadvantaged populations, where preventive services and oral health education are often lacking.

Several behavioral and contextual factors—such as poor brushing habits, high sugar intake, low parental education, and orthodontic appliances—have been consistently associated with gingival inflammation in youth (5, 9, 11). Although various studies have examined these determinants individually, no recent synthesis has focused specifically on gingivitis in pediatric populations across diverse regions.

This narrative review aims to provide an updated overview of the epidemiology and risk factors of gingivitis in children and adolescents, and to support the development of targeted preventive strategies in clinical and public health contexts.

## Methods

This narrative review was conducted to synthesize recent epidemiological data on gingivitis in children and adolescents, and to identify associated sociodemographic, behavioral, and clinical risk factors. The review followed PRISMA-ScR guidelines for scoping literature reviews.

A structured search was performed in PubMed, Scopus, and Google Scholar to identify relevant peer-reviewed articles published in English and French between January 2014 and March 2024. The search strategy combined terms such as “gingivitis,” “children,” “adolescents,” “prevalence,” “epidemiology,” “risk factors,” and “oral hygiene,” using Boolean operators (AND/OR). Only articles reporting epidemiological data on gingivitis among children and/or adolescents were considered. Systematic reviews, cross-sectional studies, and populationbased surveys were eligible.

Studies were included if they:

- Focused on individuals aged 6–18 years;
- Reported gingivitis prevalence or associated risk factors;
- Were available in full text and published in English or French;
- Concerned plaque-induced gingivitis.

Studies were excluded if they:

- Focused exclusively on individuals aged 18 years and older;
- Addressed periodontitis, genetic syndromes, or histological mechanisms without epidemiological data;
- Were not accessible in full text.

**Figure 1:**
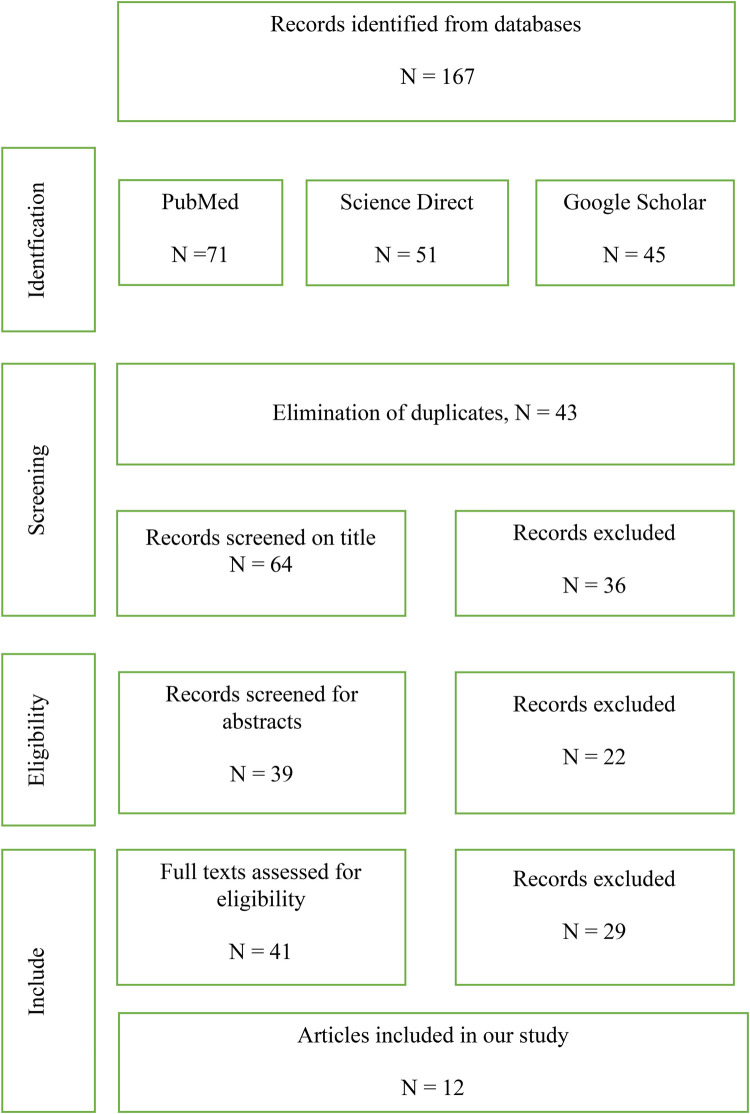
PRISMA flow diagram illustrating the study selection process.

No statistical meta-analysis or power calculation was performed, as the aim was to qualitatively synthesize the findings. Data were extracted and grouped according to prevalence rates, identified risk factors, and geographic or socioeconomic contexts (Figure 1).

## Results

This review included 12 studies published between 2014 and 2024, conducted in various regions including Africa, Asia, and Europe, and involving pediatric populations aged 6–18 years.

The prevalence of gingivitis reported in these studies ranged from approximately 20% to over 90%, depending on the age group, diagnostic criteria, and study location (4–6, 9, 13).

The highest prevalence rates were consistently found in low- and middle-income countries, where limited access to preventive dental care, poor oral hygiene habits, and lack of public health programs are prevalent (5–7, 10).

Adolescents, particularly those around puberty, appeared more susceptible to gingival inflammation—likely due to hormonal changes that modulate immune response in the gingival tissues (1–9).

Across the reviewed studies, the most commonly reported risk factors included:

- Poor oral hygiene practices, including inadequate toothbrushing and infrequent dental visits (4–6),
- Low socioeconomic status and low parental education (5–11),
- Frequent sugar intake and unbalanced diet (8–12),
- Orthodontic appliances and dental malocclusions, which favor plaque retention (9, 10),
- Lifestyle and geographical disparities, such as urban vs. rural settings, brushing frequency, and gender differences (8, 13, 14).

Most studies classified cases as mild gingivitis, characterized by redness, bleeding, and gingival swelling. However, multiple authors highlighted that unmanaged cases may progress to more severe periodontal disease in adulthood (3–12).

No quantitative meta-analysis or statistical comparison was conducted due to heterogeneity in study design and outcome measures (Table 1).

**Table 1:** Comparative table of studies on gingivitis in children and adolescents.

Author/year	Country	Population	Prevalence	Risk factors
Davidovich et al., 2020 (2)	Israel	2.5–7 y/o	Comparative study: moderate-severe gingivitis 1.8× more frequent in children with malocclusions	Effective toothbrushing and plaque control. Malocclusions increase moderate-to-severe gingivitis risk.
Folayan et al., 2021 (3)	Nigeria	10–19 y/o	57% mild, 7.8% moderate, 0.7% severe	Frequent intake of refined carbohydrates strongly linked to gingivitis. Socioeconomic and family influences.
Liu et al., 2022 (4)	China	6–12 y/o	46.6% (Sichuan), 29.1% (Shandong)	Low parental education, limited dental care access, poor-quality oral hygiene products, and unhealthy diets.
Shah et al., 2021 (5)	India	6–12 y/o	99.6%	High sugar and carbohydrate intake encourages bacterial growth. Age-related risk (10 y/o peak)
Tankova et al., 2021 (6)	Bulgaria	10–14 y/o	88% mild	Orthodontic anomalies, defective restorations
Folayan et al., 2022 (7)	Nigeria	6–11 y/o	63.3%	Low socioeconomic status (SES) correlates with higher prevalence.
Kyaw Myint et al., 2020 (8)	Myanmar	10–11 y/o	High prevalence	Low brushing frequency. Daily consumption of sweet drinks. High OHI-S scores (poor oral hygiene). High bacterial plaque levels. Parental socioeconomic status (SES).
Fan et al., 2021 (9)	China	12–15 y/o	29.6%	Poor brushing habits, infrequent dental visits, rural residence, and normalization of bleeding gums.
AlGhamdi et al., 2020 (10)	Saudi Arabia	15–19 y/o	85.6%	Barriers to dental care (financial, linguistic, service availability) among non-Saudi youth raise prevalence.
Holubieva et al., 2024 (11)	Ukraine	Children/adolescents	89,7%	Poor oral hygiene, dentoalveolar anomalies, carious cavities and proximal restorations, irregular toothbrushing and poor interdental hygiene.
Olczak-Kowalczyk et al., 2024 (12)	Poland	3–7 y/o	12,25%	Early childhood factors, plaque influenced by socioeconomic, oral hygiene, and dietary factors.
Mazzoleni et al., 2020 (13)	Global review	Children/adolescents	≥70% after age 7	Hormonal changes, poor oral hygiene

## Discussion

Gingivitis in children and adolescents is a highly prevalent and preventable periodontal disease that reflects both individual behaviors and broader structural determinants. This discussion synthesizes the epidemiological patterns, risk factors, and preventive strategies identified in the literature to better inform future public health interventions.

### Prevalence and patterns of disease

The reviewed studies consistently report a high, yet heterogeneous, prevalence of gingivitis among pediatric populations. This variability is attributed to differences in diagnostic criteria, age ranges, and geographical locations (4–6, 9, 13–14). Notably, gingival inflammation tends to increase with age, reaching a peak during adolescence, likely due to hormonal changes that enhance the gingival inflammatory response (1–9). Disparities in disease burden are particularly pronounced in low- and middle-income settings, where socioeconomic inequalities limit access to dental services and preventive care (5–7, 10)

### Contributing risk factors

While the accumulation of dental plaque remains the primary etiological factor in gingivitis, multiple aggravating conditions influence its severity and progression. Poor oral hygiene practices, such as inadequate toothbrushing and infrequent dental visits, are consistently associated with increased gingival inflammation (4–6, 11). Socioeconomic disadvantage is a significant predictor, encompassing limited oral health education, dietary imbalances, and poor access to preventive services (5, 11, 12).

Orthodontic conditions and defective restorations also play a role by creating niches that promote plaque accumulation and hinder effective cleaning (9–13). Behavioral factors—including excessive consumption of refined sugars, adolescent smoking, and psychosocial stress—have been linked to higher susceptibility and severity of gingival disease (6–8).

### Orthodontic and restorative considerations

Adolescents undergoing orthodontic treatment are particularly vulnerable to gingivitis. Fixed appliances such as brackets and wires complicate oral hygiene maintenance, leading to increased plaque retention and inflammation (10–13). Similarly, defective restorations can act as plaque-retentive factors, emphasizing the need for careful dental material selection and maintenance in pediatric patients (9).

### Prevention and management strategies

A consensus across studies highlights the critical importance of early and sustained prevention. Effective daily oral hygiene—particularly twice-daily brushing with fluoridated toothpaste and flossing—is the cornerstone of gingivitis control (3–14). School-based oral health programs have shown promise in raising awareness and establishing positive habits among children and their families. Regular dental check-ups, combined with professional prophylaxis, enable the early identification and management of inflammation. The adjunctive use of antiseptic mouthwashes has also demonstrated efficacy in reducing bacterial load and gingival bleeding (3–13).

### Towards a multidimensional approach

Optimal gingivitis prevention requires a coordinated, multidisciplinary approach. Families are central to fostering consistent hygiene behaviors at home, while schools serve as strategic platforms for health education and routine screening. Dental professionals must tailor their interventions to the developmental and social contexts of pediatric patients, delivering care that is both preventive and culturally sensitive (3–13).

### Limitations

This review is limited by its narrative design and the heterogeneity of the included studies.

Differences in methodology, diagnostic criteria, and reporting may affect the comparability of prevalence and risk factor data. In addition, restricting the search to English-language articles published between 2020 and 2024 may have excluded relevant findings.

## Conclusions

Gingivitis remains the most common periodontal condition in children and adolescents, with prevalence ranging from 20% to over 90% depending on the population, diagnostic criteria, and access to care. The synthesis of recent literature confirms that biological changes during adolescence, behavioral habits such as inadequate toothbrushing and high sugar intake, and socioeconomic inequities are consistent drivers of disease burden. Orthodontic appliances and poor restorative dentistry further exacerbate plaque retention and inflammation, particularly in low- and middle-income countries where access to preventive care is limited.

This review emphasizes the urgent need for early, structured interventions targeting children and adolescents. Preventive strategies should prioritize school-based oral health education, parental involvement, and regular dental check-ups, complemented by the use of fluoride toothpaste and safe antimicrobial rinses. Policymakers should focus on reducing barriers to dental services and implementing culturally sensitive, community-based programs to promote equity in pediatric oral health.

Future research should explore longitudinal trends in gingivitis progression, investigate the microbiological and immunological mechanisms in youth, and evaluate the cost-effectiveness of preventive interventions at the community and school levels. By aligning clinical, educational, and public health efforts, gingivitis prevention can become an achievable goal to improve lifelong oral and systemic health outcomes in children and adolescents.
