# Correction: Gingivitis in children and adolescents: epidemiological overview and associated factors—a narrative review

**DOI:** 10.3389/froh.2025.1732833

**Published:** 2025-11-12

**Authors:** Fatima Ezzahra Elgasmi, Kenza Maghous, Bouchra Badre

**Affiliations:** 1 Department of Odontological Sciences, Faculty of Medicine, Pharmacy and Dental Medicine, Sidi Mohammed Ben Abdellah University, Fez, Morocco; 2 Private Sector, Casablanca, Morocco; 3 Department of Pediatric Dentistry and Laboratory of Community Health, Epidemiology and Biostatistics, Faculty of Dental Medicine, Hassan II University, Casablanca, Morocco

**Keywords:** gingivitis, children, adolescents, epidemiology, risk factors, oral hygiene, prevention

**Author affiliation**

Affiliation [Department of Odontological Sciences] was erroneously given as [Department of Pediatric Dentistry].

The original version of this article has been updated.

**Acknowledgments**

In the acknowledgements, [we forgot to state that The study idea originally came from Prof. Bouchra Badre, as a form of acknowledgment and academic support.].

The original version of this article has been updated.

